# Varus-valgus instability in the anterior cruciate ligament-deficient knee: effect of posterior tibial load

**DOI:** 10.1186/s40634-017-0087-3

**Published:** 2017-06-27

**Authors:** Tomoki Ohori, Tatsuo Mae, Konsei Shino, Yuta Tachibana, Hiromichi Fujie, Hideki Yoshikawa, Ken Nakata

**Affiliations:** 1 Department of Orthopaedic Surgery, Osaka University Graduate School of Medicine, 2-2, Yamada-oka, Suita, Osaka, 565-0871 Japan; 2 Sports Orthopaedic Surgery Center, Yukioka Hospital, 2-2-3, Ukita, Kita-ku, Osaka, 530-0021 Japan; 3 Intelligent Mechanical Systems, Graduate School of System Design, Tokyo Metropolitan University, 6-6, Asahiga-oka, Hino, Tokyo, 191-0065 Japan

**Keywords:** Anterior cruciate ligament, Instability, Laxity, Varus, Valgus, Posterior tibial load, Medial collateral ligament, Lateral collateral ligament, Knee

## Abstract

**Background:**

Anterior cruciate ligament (ACL) injury is often accompanied with medial collateral ligament (MCL) injury. Assessment of varus-valgus (V-V) instability in the ACL-deficient knee is crucial for the management of the concomitant ACL-collateral ligaments injury. We evaluated the V-V laxity and investigated the effect of additional posterior tibial load on the laxity in the ACL-deficient knee. Our hypothesis was that the V-V laxity in the ACL-deficient knee was greater than that in the intact knee and attenuated by additional posterior tibial load.

**Methods:**

Eight fresh-frozen porcine knees were used, and a 6°-of-freedom (DOF) robotic system was utilized. A 5 Nm of V-V torque was applied to the intact knee, the ACL-deficient knee, and the ACL-deficient knee with 30 N of constant posterior tibial load, at 30° and 60° of flexion. Then, the 3D path in the intact knee was reproduced on the ACL-deficient knee. The total V-V angle under 5 Nm of V-V torque was assessed and compared among the three statuses. The *in situ* forces of the ACL under 5 Nm of varus and valgus torques, respectively, were also calculated.

**Results:**

The total V-V angle in the ACL-deficient knee under 5 Nm of V-V torque was significantly greater than that in the intact knee, whereas the angle in the ACL-deficient knee with 30 N of posterior tibial load was significantly smaller than that in the ACL-deficient knee and approached that in the intact knee, at both 30° and 60° of flexion. The *in situ* force of the ACL was approximately 30 N at 30° and 16 N at 60° of flexion under 5 Nm of both varus and valgus torques.

**Conclusions:**

The V-V laxity in the isolated ACL-deficient knee was greater than that in the intact knee. The increased laxity was attenuated and approached that in the intact knee by adding posterior tibial load. Application of posterior tibial load is necessary for accurate assessment of V-V instability in the ACL-deficient knee. Clinically, the V-V laxity in the combined ACL-MCL or ACL-LCL injured knee may be overestimated without posterior tibial load.

## Background

Anterior cruciate ligament (ACL) injury is often associated with concomitant injury to other ligaments, especially the medial collateral ligament (MCL) (Majewski et al. 2006). Associated injury to the MCL is observed in 4–17% of all patients with ACL injury (Kaeding et al. 2005; Shelbourne & Nitz 1991). Although most MCL injuries heal conservatively with early functional rehabilitation (Holden et al. 1983; Indelicato 1983; Petermann et al. 1993; Reider et al. 1993), some cases with concomitant grade III MCL injury need surgical treatment (Fetto & Marshall 1978; Hillard-Sembell et al. 1996; Hughston 1994; Kovachevich et al. 2009; Shelbourne & Porter 1992; Wijdicks et al. 2010). Grant et al. (2012) recommended MCL repair or reconstruction in case of persistent valgus instability after conservative treatment. Lateral collateral ligament (LCL) injury is also combined with ACL injury, while the frequency of the ACL-LCL injury is lower than that of the ACL-MCL injury (Majewski et al. 2006). LCL repair or reconstruction is needed when varus instability remains in the ACL-reconstructed knee (LaPrade et al. 1999). Therefore, assessment of varus-valgus (V-V) instability in the ACL-deficient knee is critical, as persistent V-V instability with ACL injury is an indication for operative treatment either prior to or concomitantly with ACL reconstruction. Magnetic resonance imaging (MRI) may help in the diagnosis of collateral ligaments injury. However, MRI grading did not correspond to clinical grading in some cases with MCL (Halinen et al. 2009; Jacobson et al. 2006; Schweitzer et al. 1995) and LCL (Bonadio et al. 2014) injury. Thus, physical examination for the assessment of V-V instability in the ACL-deficient knee is crucial for the management of these patients.

ACL deficiency may affect the V-V laxity in the human knee joint (Imbert et al. 2014; Imbert et al. 2015; Markolf et al. 1984). Markolf et al. (Markolf et al. 1984) reported a 36% increase in the V-V laxity at full extension in the isolated ACL-deficient knee on application of 20 Nm of V-V torque. Therefore, the V-V laxity in patients with injury to both the ACL and the MCL can be much greater than that with isolated MCL injury. Additionally, the anterior tibial translation in the ACL-deficient knee was shown to be greater than that in the intact knee, even under non-weight bearing conditions (DeFrate et al. 2006; Matsuo et al. 2014; Mishima et al. 2005). We postulated that anterior tibial translation might be associated with greater V-V laxity in the ACL-deficient knee. Therefore, the objectives of this study were 1) to evaluate the V-V laxity in the isolated ACL-deficient knee on application of V-V torque and 2) to clarify the effect of additional posterior tibial load on the laxity in the ACL-deficient knee. We hypothesized that the V-V laxity in the isolated ACL-deficient knee under V-V torque was greater than that in the intact knee, and that the laxity in the isolated ACL-deficient knee was attenuated by adding posterior tibial load.

## Methods

Eight fresh-frozen porcine knees were used in this study. Their mean age and weight was approximately 24 weeks (range, 23–25) and 115 kg (110–120), respectively. Each knee was thawed at room temperature for 24 h prior to testing. The patella, patellar tendon and all muscles except for the popliteus were removed, while the capsule around the knee was carefully left. Knees with apparent injury to ligaments, menisci, and cartilage of the articular surface were excluded. The femur and the tibia were cut 13 cm apart from the joint line, and both ends were potted and fixed in cylindrical molds of acrylic resin (Ostron II; GC, Tokyo, Japan). The fibula was cut 4 cm distal from the proximal tibiofibular joint and fixed in its anatomic position with acrylic resin.

### Apparatus

A 6°-of-freedom (DOF) robotic system was utilized in the study. The system consisted of a velocity-control 6-axis manipulator (custom-designed) with a universal force/moment sensor (UFS) (SI–660–60, ATI Industrial Automation, NC, USA) and a control computer (Windows XP; Microsoft, WA, USA) linked with a high-speed motion network (Mechatrolink-II; Yaskawa Electric, Fukuoka, Japan) (Fujie et al. 1993; Fujie et al. 1996; Fujie et al. 2004). The manipulator had two mechanisms: the upper mechanism attached to the UFS and the lower one. The upper mechanism was linked to two translational-axis actuators (SGDS-01F12A; Yaskawa Electric, Fukuoka, Japan) and three rotational-axis actuators (HA–800B–3A; Harmonic Drive Systems, Tokyo, Japan), while the lower one was linked to one translational-axis actuator. All the actuators were powered by AC servo-motors. The control computer in a graphical language programming environment (LabView 8.6.1; National Instruments, TX, USA) operated the program to control both the position of and the force/moment acting on the knee joint. The system could manipulate a natural three-dimensional (3D) motion of the knee, prescribing the force/moment acting on a joint except for the operator’s intended direction at zero, by calculating acquired data of the position and the force/moment. The manipulator had a position accuracy of ± 0.003 mm in translation and ± 0.002° in rotation; the clamp-to-clamp stiffness was more than 319 N/mm in translation and more than 84.6 Nmm/° in rotation (Fujie et al. 2013). Iteration of data acquisition, kinematic and kinetic calculation, and motion of actuator were performed at a rate of 20 Hz.

**Fig. 1:**
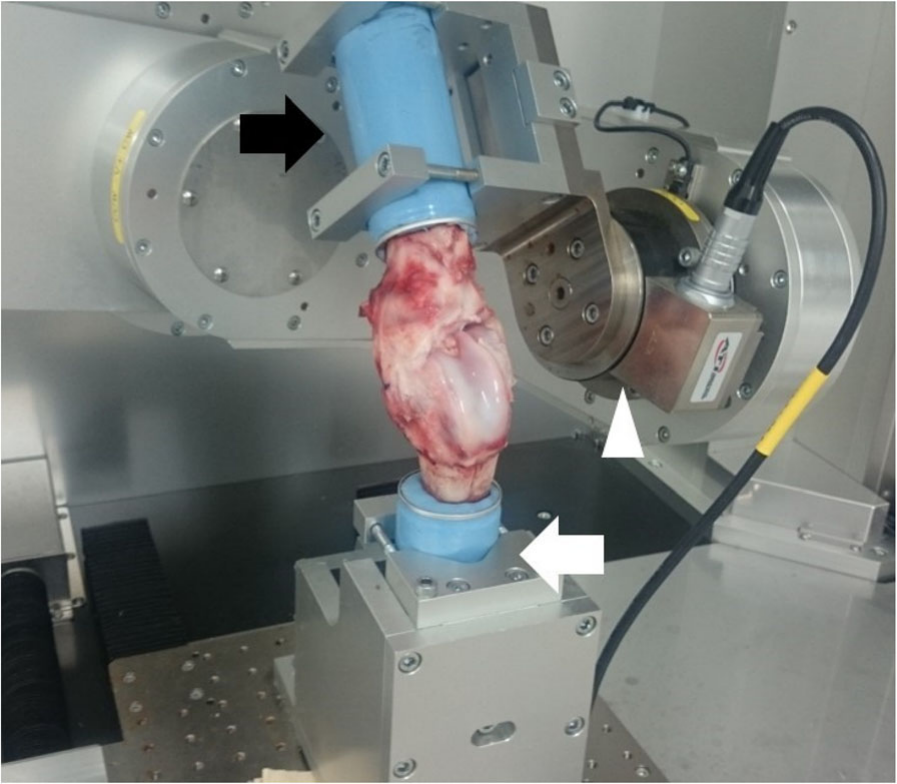
The 6°-of-freedom (DOF) robotic system. The tibial cylindrical molded end was connected to the upper mechanism of 6-axis manipulator (black arrow), while the femoral end was connected to the lower one (white arrow). The white arrow head indicates the universal force/moment sensor (UFS) attached to the upper mechanism

The tibial cylindrical molded end was connected firmly to the upper mechanism of the manipulator via a specially designed aluminum clamp, while the femoral end was connected firmly to the lower one (Fig. 1). A knee joint coordinate system developed by Grood and Suntay (Grood & Suntay 1983) was introduced, and a 3D digitizer (MicroScribe-3DX; Immersion, CA, USA) was utilized to aim the femoral insertion sites of the MCL and LCL (resolution: 0.13 mm; accuracy: 0.23 mm).

### Testing protocol

At the beginning of the examination, three times of passive flexion-extension motion between 20° and 120° of flexion were applied to the intact knee to exclude the influence of creep behavior. In the third cycle, the internal-external (I-E) rotational positions at 30° and 60° of flexion were recorded, respectively; these were considered as natural I-E rotational positions in the intact knee.

**Fig. 2:**
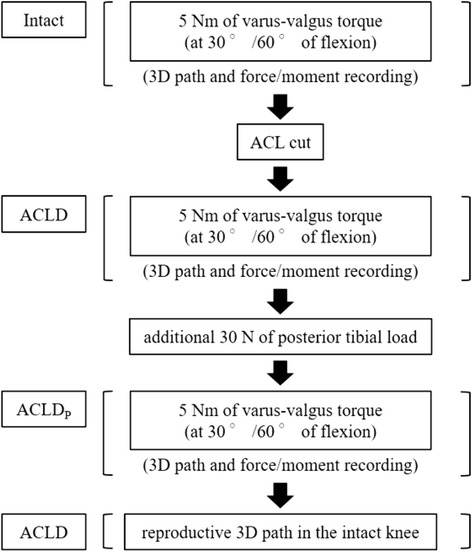
Testing protocol. All procedures were carried out under fixation of the natural internal-external rotational positions at 30° and 60° of flexion, respectively. *Intact*: intact knee, *ACLD*: ACL-deficient knee, *ACLD*_*P*_: ACL-deficient knee with additional 30 N of posterior tibial load

First, a 5 Nm of V-V torque was loaded on the intact knee at 30° and 60° of flexion, respectively, and the 3D path and the force/moment of the tibia relative to the femur were recorded. After cutting the ACL, the same procedure was followed for the ACL-deficient knee, and the 3D path and the force/moment were recorded. In the pilot study, we evaluated the optimal posterior tibial load to maintain the normal femur-tibial position in the ACL-deficient knees, and decided that 30 N was enough to return the anterior tibial displacement to the neutral position. Then, with additional 30 N of constant posterior tibial load, the same procedure was performed on the ACL-deficient knee, and the 3D path and the force/moment were recorded. Finally, the previously recorded 3D path in the intact knee was reproduced on the ACL-deficient knee (Fig. 2). All procedures were carried out under fixation of the natural I-E rotational positions at 30° and 60° of flexion, respectively, in accordance with the situation of clinical assessment.

We assessed the total V-V angle under 5 Nm of V-V torque, and compared the V-V angle among the intact knee, the ACL-deficient knee, and the ACL-deficient knee with additional posterior tibial load. The varus and valgus angles and the related anterior tibial translations from the neutral position were also evaluated under 5 Nm of varus and valgus torques, respectively. The *in situ* forces of the ACL under 5 Nm of varus and valgus torques, respectively, were calculated from the tibial force/moment data, under the principle of superposition (Fujie et al. 1995; Fujie et al. 2004).

### Statistical analysis

All statistical analyses were performed with JMP software (JMP Pro version 12.0.2; SAS Institute, NC, USA). Power analysis (power 0.8; α 0.05) indicated a sample size requirement of seven knees for valid comparisons. The Steel-Dwass test for multiple comparisons was used to assess between-group differences. A *p*-value < 0.05 was considered statistically significant.

## Results

### Total varus-valgus angle and related anterior tibial translation

On application of 5 Nm of V-V torque, the total V-V angle in the ACL-deficient knee was significantly greater than that in the intact knee at both 30° and 60° of flexion (*p* = 0.01). The angle in the ACL-deficient knee with 30 N of posterior tibial load was significantly smaller than that in the ACL-deficient knee (*p* = 0.01) and approached that in the intact knee at both 30° and 60° of flexion (Table 1). The varus angle was significantly different among the three knee models, while the valgus angle did not show any difference.

**Table 1:** Total varus-valgus angle and varus and valgus angles, respectively, under 5 Nm of varus-valgus torque

	Intact	ACLD	ACLD_p_
Total varus-valgus angle at 30° of flexion (°)	6.9 ± 1.1	8.7 ± 1.5 ^a^	7.4 ± 1.5
Varus angle (°)	3.6 ± 0.5	5.0 ± 0.8 ^a^	3.7 ± 0.9
Valgus angle (°)	3.3 ± 1.0	3.6 ± 1.1	3.7 ± 1.0
Total varus-valgus angle at 60° of flexion (°)	8.7 ± 1.5	10.7 ± 2.6 ^a^	8.9 ± 2.2
Varus angle (°)	3.8 ± 2.4	5.7 ± 3.6 ^a^	3.8 ± 3.0
Valgus angle (°)	4.9 ± 2.3	5.0 ± 2.4	5.0 ± 2.3

Mean ± standard deviation, ^a^ significant difference compared to the values in the Intact and the ACLD_p_ (*p* = 0.01)

*Intact* intact knee, *ACLD* ACL-deficient knee, *ACLD*_*P*_ ACL-deficient knee with additional 30 N of posterior tibial load

The anterior tibial translation in response to 5 Nm of varus torque in the ACL-deficient knee was significantly greater than that in the intact knee at both 30° and 60° of flexion (*p* = 0.01), and the anterior tibial translation in the ACL-deficient knee with 30 N of posterior tibial load was significantly smaller than that in the ACL-deficient knee (*p* = 0.01) (Table 2). On the other hand, the anterior tibial translation under 5 Nm of valgus torque represented no significant difference among the three groups.

**Table 2:** Anterior tibial translation under 5 Nm of varus and valgus torques, respectively

	Intact	ACLD	ACLD_p_
Anterior tibial translation at 30° of flexion (mm)			
Under varus torque	+1.6 ± 0.7	+4.0 ± 0.9 ^a^	0.0 ± 1.2
Under valgus torque	–0.8 ± 0.9	–0.8 ± 1.3	– 2.2 ± 1.4
Anterior tibial translation at 60° of flexion (mm)			
Under varus torque	+2.2 ± 1.2	+3.9 ± 2.2 ^a^	+0.3 ± 1.6
Under valgus torque	– 1.5 ± 1.4	– 1.9 ± 1.5	– 3.0 ± 1.7

Mean ± standard deviation, the positive value indicates anterior tibial translation, and the negative one does posterior translation, ^a^ significant difference compared to the values in the Intact and the ACLD_p_ (*p* = 0.01)

*Intact*: intact knee, *ACLD*: ACL-deficient knee, *ACLD*_*P*_: ACL-deficient knee with additional 30 N of posterior tibial load

### *In situ* force of the ACL

The *in situ* force of the ACL increased in direct proportion to the amount of torque on application of V-V torque, and was approximately 30 N at 30° and 16 N at 60° of flexion under 5 Nm of both varus and valgus torques (Table 3).

**Table 3:** The values of *in situ* force of the ACL under 5 Nm of varus and valgus torques, respectively

*In situ* force (N)	Under 5 Nm of varus torque	Under 5 Nm of valgus torque
At 30 ° of flexion	27.8 ± 14.0	32.4 ± 15.7
At 60 ° of flexion	16.4 ± 12.0	15.9 ± 6.1

## Discussion

The principal findings of the present study were that the V-V laxity in the ACL-deficient knee in response to V-V torque was greater than that in the intact knee, and that the laxity in the ACL-deficient knee with additional posterior tibial load got close to that in the intact knee. Therefore, the ACL appeared to carry a substantial role in restraining V-V rotation of the knee joint.

As the ACL worked against anterior tibial drawer, the anterior tibial translation under anterior tibial load increased in the ACL-injured knee (Amis & Dawkins 1991; Markolf et al. 1984). Amis et al. (1991) reported that the anterior tibial translation increased by approximately 140% after removal of the ACL on application of 150 N of anterior tibial load at 20° of flexion. Besides, Markolf et al. (1984) reported the increased V-V laxity in the ACL-deficient knee in response to V-V torque, as compared to that in the intact knee. Our results showed that the V-V laxity increased under V-V torque, while the *in situ* force of the ACL was 16–30 N under 5 Nm of varus and valgus torques, respectively. Thus, the ACL also had a restraining force against V-V rotation of the knee joint (Grood et al. 1981; Markolf et al. 1990). In addition, the tibia in the ACL-deficient knee located more anteriorly than in the intact knee even under non-weight bearing conditions (DeFrate et al. 2006; Matsuo et al. 2014; Mishima et al. 2005). Matuso et al. (2014) reported that the anterior tibial translation in the ACL-deficient knee was 1.4 mm greater than that in the normal knee in supine and extended knee position. Hence, the anterior tibial translation was assumed to be associated with the greater V-V laxity in the ACL-deficient knee. In the present study, the increase in V-V laxity in the ACL-deficient knee was attenuated by adding posterior tibial load because the anterior tibial translation was restrained. These findings suggest that posterior tibial load should be applied during assessment of V-V instability in the ACL-deficient knee for accurate physical evaluation of the V-V laxity.

**Fig. 3:**
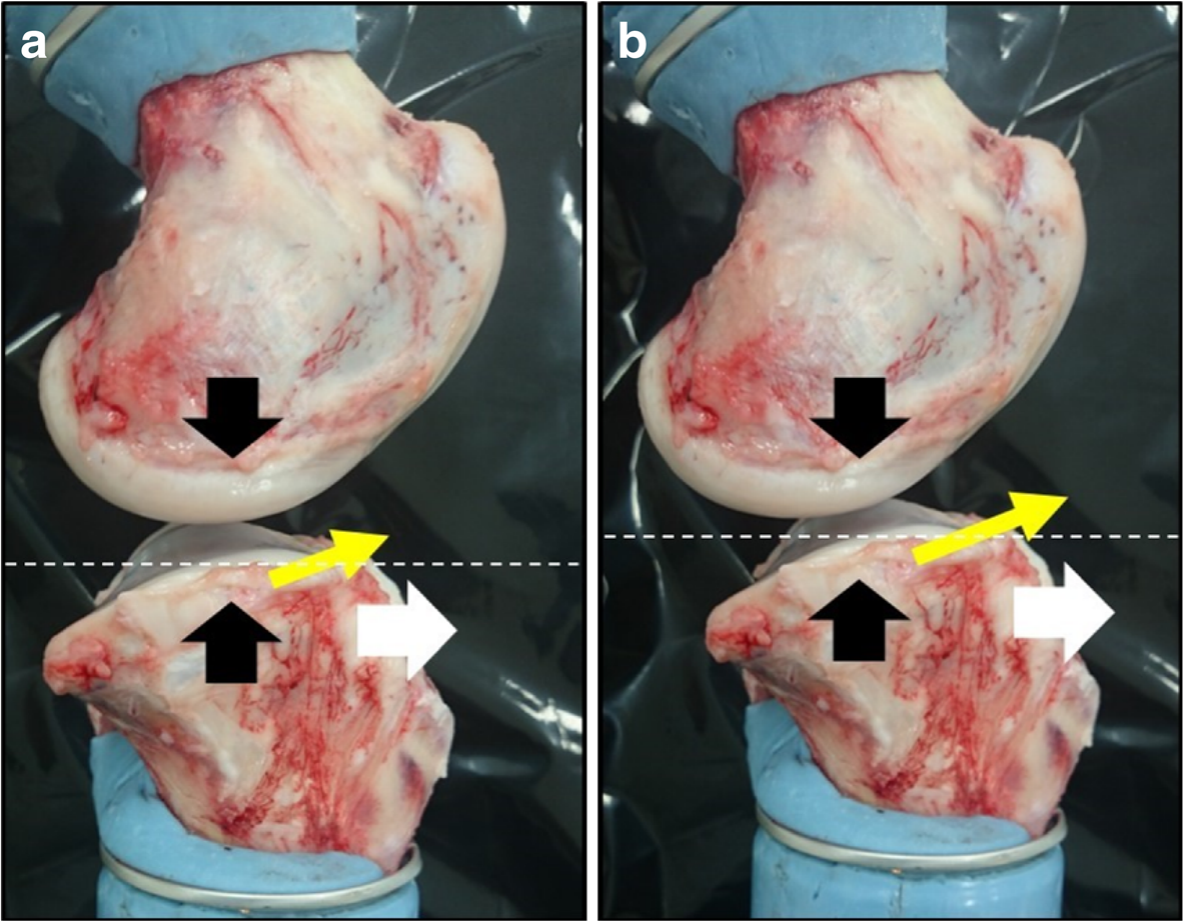
Lateral view of porcine knee joint (medial side). **a** when varus torque is applied, the interaction between the medial femoral condyle and the medial tibial plateau (*black arrow*) generates anterior tibial load (white arrow) and the tibia translates in antero-proximal direction (*yellow arrow*) along the posterior tibial slope, **b** the tibia can rotate in more varus direction because of the greater antero-proximal translation after removal of the ACL (white dotted line)

**Fig. 4:**
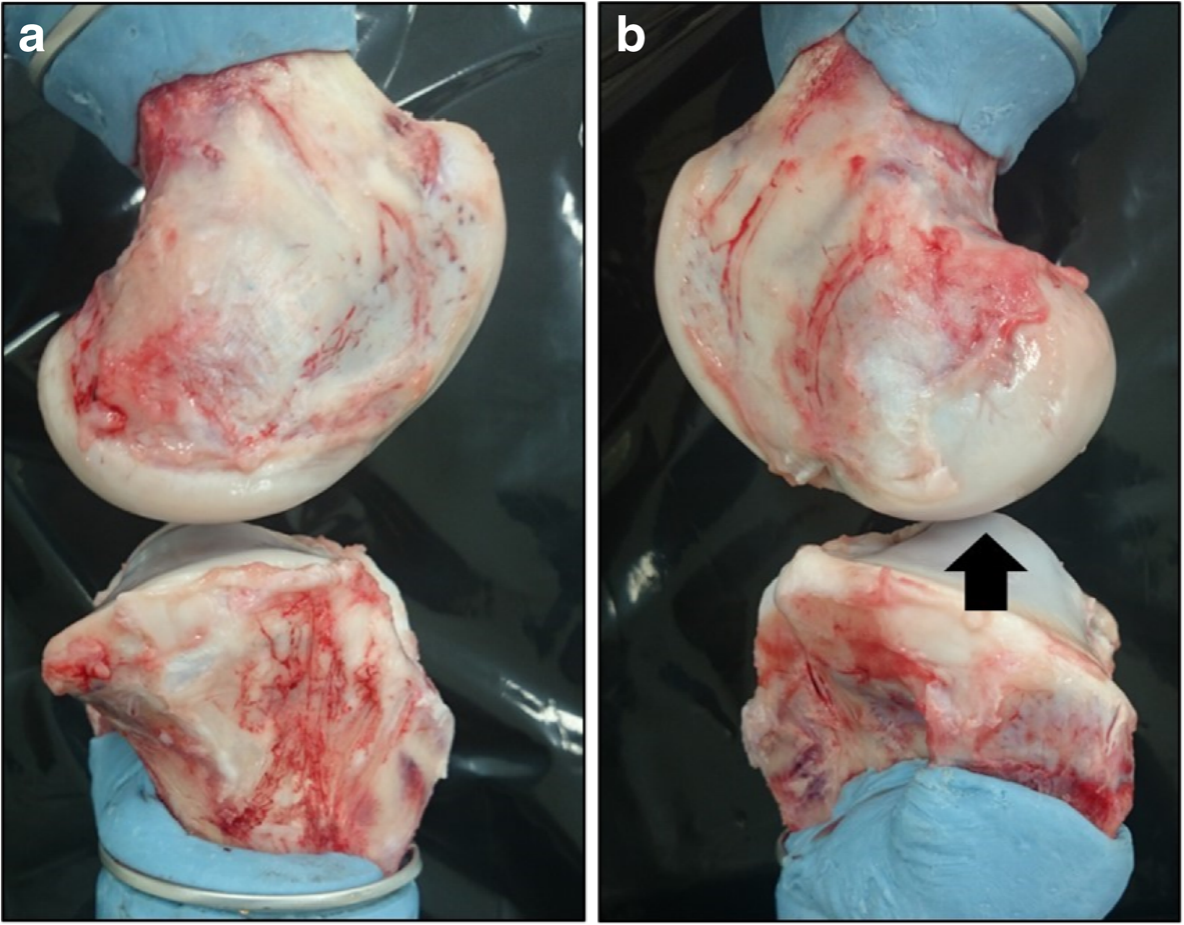
Lateral view of porcine knee joint [medial (**a**) and lateral (**b**) side]. The anterior slope of the lateral tibial convex plateau seemed to be too steep (black arrow) to overcome the lateral femoral condyle and translate anterio-proximally in response to valgus torque under restriction of the internal rotation

When varus torque is applied to the knee joint, the interaction between the medial femoral condyle and the medial tibial plateau generates anterior tibial load due to the posterior tibial slope. This anterior load and the compressive load in the medial compartment induce the tibial translation in antero-proximal direction along the posterior tibial slope (Fig. 3a). After resection of the ACL, the tibia translates more antero-proximally than that in the intact knee because restraining force against anterior drawer is lost. Consequently, this enables the tibia to rotate in more varus direction (Fig. 3b). Our results demonstrated that the anterior tibial translation as well as the varus angle under varus torque in the ACL-deficient knee increased compared to that in the intact knee. However, this was not demonstrable for the valgus laxity. On macroscopic observation, the anterior slope of the lateral tibial convex plateau seemed to be very steep; therefore, the tibia might not be able to overcome the lateral femoral condyle and translate antero-proximally in response to valgus torque, under fixation of the I-E rotation of the knee joint (Fig. 4). Fukuda et al. (2003) reported that under conditions of unfixed I-E rotation, valgus torque caused the greater anterior tibial translation coupled with the internal rotation in the ACL-deficient knee, as compared to the intact knee. In this study, as the I-E rotation of the knee joint was restricted in order to mimic the assessment in clinical settings, this condition might affect the valgus rotation.

There were some limitations in the present study. First, we used porcine knee model. However, porcine knee has been proved to be anatomically similar to human knee and available for biomechanical investigations (Aerssens et al. 1998; Boquszewski et al. 2011; Martin et al. 2016). So, the results obtained from this study can be applied to the clinical assessment of human knee joint reasonably well. Second, removal of the patella and the patellar tendon might lead to overestimation of the V-V laxity in the ACL deficient knee (Guenther et al. 2016; Thein et al. 2016). However, the influence on the measured laxity could be minimal because the anteromedial and anterolateral capsule were carefully left. Finally, the experiment was performed under conditions of fixed I-E rotation (4-DOF). Inoue et al. (1987) reported overestimation of the V-V laxity in the isolated ACL-deficient canine knee was in 5-DOF mode, as compared to that in 3-DOF mode (fixation of I-E rotation and anterior-posterior translation), due to the coupled I-E tibial rotation under V-V torque. When we clinically assess V-V instability of the knee joint, the I-E rotation is usually restricted. Therefore, we assessed the V-V laxity in 4-DOF manner for more precise measurements.

## Conclusions

The V-V laxity in the isolated ACL-deficient knee increased compared to that in the intact knee under V-V torque. The increased laxity was attenuated and got close to that in the intact knee by adding posterior tibial load. Therefore, it is necessary to apply posterior tibial load against the anterior tibial translation for accurate assessment of V-V instability in the ACL-deficient knee.

## Abbreviations

3D: Three-dimensional
ACL: Anterior cruciate ligament
DOF: Degree-of-freedom
I-E: Internal-external
LCL: Lateral collateral ligament
MCL: Medial collateral ligament
MRI: Magnetic resonance imaging
UFS: Universal force/moment sensor
V-V: Varus-valgus

## References

[CR1] Aerssens J, Boonen S, Lowet G, Dequeker J (1998). Interspecies differences in bone composition, density, and quality: potential implications for in vivo bone research. Endocrinology.

[CR2] Amis AA, Dawkins GP (1991). Functional anatomy of the anterior cruciate ligament. Fibre bundle actions related to ligament replacements and injuries. J Bone Joint Surg (Br).

[CR3] Bonadio MB, Helito CP, Gury LA, Demange MK, Pécora JR, Angelini FJ (2014). Correlation between magnetic resonance imaging and physical exam in assessment of injuries to posterolateral corner of the knee. Acta Orthop Bras.

[CR4] Boquszewski DV, Shearn JT, Wagner CT, Butler DL (2011). Investigating the effects of anterior tibial translation on anterior knee force in the porcine model: is the porcine knee ACL dependent?. J Orthop Res.

[CR5] DeFrate LE, Papannagari R, Gill TJ, Moses JM, Pathare NP, Li G (2006). The 6° of freedom kinematics of the knee after anterior cruciate ligament deficiency: an in vio imaging analysis. Am J Sports Med.

[CR6] Fetto JF, Marshall JL (1978). Medial collateral ligament injuries of the knee: a rationale for treatment. Clin Orthop Relat Res.

[CR7] Fujie H, Mabuchi K, Woo SL, Livesway GA, Arai S, Tsukamoto Y (1993). The use of robotics technology to study human joint kinematics: a new methodology. J Biomech Eng.

[CR8] Fujie H, Livesay GA, Woo SL, Kashiwaguchi S, Blomstrom G (1995). The use of a universal force-moment sensor to determine in-situ forces in ligaments: a new methodology. J Biomech Eng.

[CR9] Fujie H, Livesay GA, Fujita M, Woo SL (1996). Forces and moments in six-DOF at the human knee joint: mathematical description for control. J Biomech.

[CR10] Fujie H, Sekito T, Orita A (2004). A novel robotic system for joint biomechanical tests: application to the human knee joint. J Biomech Eng.

[CR11] Fujie H, Kimura K, Yamakawa S (2013) Static and dynamic properties of a 6-DOF robotic system for knee joint biomechanics study. In: Transections of the ASME 2013 Summer Bioengineering Conference, Sun River, Oregon, 26–29 June 2013: 14849

[CR12] Fukuda Y, Woo SL, Loh JC, Tsuda E, Tang P, McMahon PJ, Debski RE (2003). A quantitative analysis of valgus torque on the ACL: a human cadaveric study. J Orthop Res.

[CR13] Grood ES, Noyes FR, Butler DL, Suntay WJ (1981). Ligamentous and capsular restraints preventing straight medial and lateral laxity in intact human cadaver knees. J Bone Joint Surg Am.

[CR14] Grood ES, Suntay WJ (1983). A joint coordinate system for the clinical description three-dimensional motions: application to the knee. J Biomech Eng.

[CR15] Grant JA, Tannenbaum E, Miller BS, Bedi A (2012). Treatment of combined complete tears of the anterior cruciate ligament and medial collateral ligaments. Arthroscopy.

[CR16] Guenther D, Rahnemai-Azar AA, Bell KM, Irarrázaval S, Fu FH, Musahl V, Debski RE (2016). The anterolateral capsule of the knee behaves like a sheet of fibrous tissue. Am J Sports Med.

[CR17] Halinen J, Koivikko M, Lindahl J, Hirvensalo E (2009). The efficacy of magnetic resonance imaging in acute multi-ligament injuries. Int Orthop.

[CR18] Hillard-Sembell D, Daniel DM, Stone ML, Dobson BE, Fithian DC (1996). Combined injuries of the anterior cruciate and medial collateral ligaments of the knee. Effect of treatment on stability and function of the joint. J Bone Joint Surg Am.

[CR19] Holden DL, Eggert AW, Butler JE (1983). The nonoperative treatment of grade I and II medial collateral ligament injuries to the knee. Am J Sports Med.

[CR20] Hughston JC (1994). The importance of the posterior oblique ligament in repairs of acute tears of the medial ligaments in knees with and without an associated rupture of the anterior cruciate ligament. Results of long-term follow-up. J Bone Joint Surg Am.

[CR21] Imbert P, Belvedere C, Leardini A (2014). Human knee laxity in ACL-deficient and physiological contralateral joints: intra-operative measurements using a navigation system. Biomed Eng Online.

[CR22] Imbert P, Belvedere C, Leardini A (2015). Knee laxity modifications after ACL rupture and surgical intra- and extra-articular reconstructions: intra-operative measures in reconstructed and healthy knees. Knee Surg Sports Traumatol Arthrosc.

[CR23] Indelicato PA (1983). Non-operative treatment of complete tears of the medial collateral ligament of the knee. J Bone Joint Surg Am.

[CR24] Inoue M, McGurk-Burleson E, Hollis JM, Woo SL (1987). Treatment of the medial collateral ligament injury. I: the importance of anterior cruciate ligament on the varus-valgus knee laxity. Am J Sports Med.

[CR25] Jacobson KE, Frederic SC (2006). Evaluation and treatment of medial collateral ligament and medial-sided injuries of the knee. Sports Med Arthrosc.

[CR26] Kaeding CC, Pedroza AD, Parker RD, Spindler KP, McCatry EC, Andrish JT (2005). Intra-articular findings in the reconstructed multiligament-injured knee. Arthroscopy.

[CR27] Kovachevich R, Shah JP, Arens AM, Stuart MJ, Dahm DL, Levy BA (2009). Operative management of the medial collateral ligament in the multi-ligament injured knee: an evidence-based systematic review. Knee Surg Sports Traumatol Arthrosc.

[CR28] LaPrade RF, Resig S, Wentorf F, Lewis JL (1999). The effects of grade III posterolateral knee complex injuries on anterior cruciate ligament graft force. A biomechanical analysis. Am J Sports Med.

[CR29] Majewski M, Susanne H, Klaus S (2006). Epidemiology of athletic knee injuries: a 10-year study. Knee.

[CR30] Markolf KL, Kochan A, Amstutz HC (1984). Measurement of knee stiffness and laxity in patients with documented absence of the anterior cruciate ligament. J Bone Joint Surg Am.

[CR31] Markolf KL, Gorek JF, Kabo JM, Shapiro MS (1990). Direct measurement of resultant forces in the anterior cruciate ligament: an in-vitro study performed with a new experimental technique. J Bone Joint Surg Am.

[CR32] Martin RK, Gillis D, Leiter J, Shantz JS, MacDonald P (2016). A porcine knee model is valid for use in the evaluation of arthroscopic skills: a pilot study. Clin Orthop Relat Res.

[CR33] Matsuo T, Mae T, Shino K, Kita K, Tachibana Y, Sugamoto K, Yoshikawa H, Nakata K (2014). Tibiofemoral relationship following anatomic triple-bundle anterior cruciate ligament reconstruction. Knee Surg Sports Traumatol Arthrosc.

[CR34] Mishima S, Takahashi S, Kondo S, Ishiguro N (2005). Anterior tibial subluxation in anterior cruciate ligament-deficient knees: quantification using magnetic resonance imaging. Arthroscopy.

[CR35] Petermann J, von Garrel T, Gotzen L (1993). Non-operative treatment of acute medial collateral ligament lesions of the knee joint. Knee Surg Sports Traumatol Arthrosc.

[CR36] Reider B, Sathy MR, Talkington J, Blyznak N, Kollias S (1993). Treatment of isolated medial collateral ligament injuries in athletes with early functional rehabilitation. A five-year follow-up study. Am J Sports Med.

[CR37] Shelbourne KD, Nitz PA (1991). The O’Donoghue triad revisited: combined knee injuries involving anterior cruciate and medial collateral ligament tears. Am J Sports Med.

[CR38] Shelbourne KD, Porter DA (1992). Anterior cruciate ligament-medial collateral ligament injury: nonoperative management of medial collateral ligament tears with anterior cruciate ligament reconstruction. A preliminary report. Am J Sports Med.

[CR39] Schweitzer ME, Tran D, Deely DM, Hume EL (1995). Medial collateral ligament injuries: evaluation of multiple signs, prevalence and location of associated bone bruises, and assessment with MR imaging. Radiology.

[CR40] Thein R, Boorman-Padgett J, Stone K, Wickiewicz TL, Imhauser CW, Pearle AD (2016). Biomechanical assessment of the anterolateral ligament of the knee: a secondary restraint in simulated tests of the pivot shift and of anterior stability. J Bone Joint Surg Am.

[CR41] Wijdicks CA, Griffith CJ, Johansen S, Engebretsen L, LaPrade RF (2010). Injuries to the medial collateral ligament and associated medial structures of the knee. J Bone Joint Surg Am.

